# An Anglocentric History of Anaesthetics and Analgesics in the Refinement of Animal Experiments

**DOI:** 10.3390/ani10101933

**Published:** 2020-10-21

**Authors:** R. Eddie Clutton

**Affiliations:** The Wellcome Trust Critical Care Laboratory for Large Animals, Roslin Institute, Easter Bush Veterinary Centre, Roslin, Midlothian EH25 9RG, UK; e.clutton@ed.ac.uk; Tel.: +44-131-650-6220 or +44-07749-887-342

**Keywords:** animal research, animal testing, biomedical research, anaesthesia, analgesia, history of science

## Abstract

**Simple Summary:**

In simultaneously describing the history of animal experimentation and the development of anaesthetics and analgesics from an Anglocentric perspective, this article reveals how the latter have considerably refined animal experiments and brought benefits to both science and the animals involved—particularly in the 19th and 20th centuries. The more recent development of training and educational programmes in laboratory animal anaesthesia and their role in maintaining desirable trends in experimental refinement are also described.

**Abstract:**

Previous histories of animal experimentation, e.g., Franco (2013) have focused on ethics, the law and the personalities involved, but not on the involvement of anaesthetics or analgesics. Given that these were major subjects of (UK) Parliamentary debates on vivisection in the mid-19th century and viewed as “indisputable refinements in animal experimentation” (Russell and Burch 1959), it seemed that an analysis of their role was overdue. This commentary has, in interweaving the history of animal experimentation in the UK with the evolution of anaesthesia, attempted to: (1) clarify the evidence for Russell and Burch’s view; and (2) evaluate anaesthesia’s ongoing contribution to experimental refinement. The history that emerges reveals that the withholding or misuse of anaesthetics and, or analgesics from laboratory animals in the UK has had a profound effect on scientists and indirectly on the attitudes of the British public in general, becoming a major driver for the establishment of the anti-vivisection movement and subsequently, the Cruelty to Animals Act (1876)—the world’s first legislation for the regulation of animal experimentation. In 1902, the mismanaged anaesthetic of a dog in the Department of Physiology, University College London resulted in numerous events of public disorder initiated by medical students against the police and a political coalition of anti-vivisectionists, trade unionists, socialists, Marxists, liberals and suffragettes. The importance of anaesthesia in animal experiments was sustained over the following 150 years as small mammalian species gradually replaced dogs and cats as the principle subjects for vivisection. In discussing experimental refinement in their 1959 report, “The Principles of Humane Experimental Technique” Russell and Burch described anaesthetics as “… the greatest single advance in humane technique, (which) has at the same time been virtually indispensable for the advance of experimental biology”. Since then, the role of anaesthetics and in particular analgesics has become an unavoidable consideration whenever animal experiments are planned and conducted. This has been accompanied by a proliferation of training and educational programmes in laboratory animal anaesthesia.

## 1. Introduction

The progression of animal experimentation from classical times to the present has been directed by the rarely compatible views of “scientists”, philosophers and legislating authorities and, more recently, vested interest and the public have become influential. Arguably, the greatest hostility between those for and against animal vivisection—based largely on the questions of animal sentience and the scientific value of noxious animal experiments—was seen in the years between the late 18th and early 19th centuries, when, co-incidentally, the first influential observations on the anaesthetic properties of gases were reported. The introduction of general anaesthetics into medical practice in the mid-19th century greatly facilitated surgery by alleviating the (human) patient’s agonies. At the same time, and for the same reason, many felt that anaesthetics increased the ethical defensibility of animal experiments. The similarly opining would also claim that in eliminating the widespread and profound physiological effects of pain and suffering—an effect which had been recognized for some 2300 years—anaesthetics improved data value and increased the validity of animal experiments. Some scientists countered this (and continue to do so) by emphasising that anaesthetics and analgesics can produce similar if not greater degrees of physiological perturbation than the pain itself, and so should be excluded from experimental protocols. Fortunately (for animals) Russell and Burch’s “The Principles of Humane Experimental Technique” (1959) [1] established anaesthesia and analgesia as indisputable refinements in animal experimentation and which, along with reduction and replacement, pervades all stages of contemporary animal experimentation in the UK, i.e., from funding application and ethical approval through to the publication of results. A focused historical examination of the contribution of anaesthesia and analgesia to the refinement of animal experiments has not previously been attempted. This article reports an Anglocentric view of the role of anaesthetics, analgesics and anaesthetists in reshaping the attitudes of scientists, philosophers, the authorities and the public to animal experimentation.

Galen, Vesalius and Paracelsus (330BC-1541)

Beginning with a description of surgical procedures conducted on animals *without* the use of anaesthetics, i.e., vivisection, reveals an early concern with its morality and an ongoing conviction that pain has a disruptive effect on experimental results. Thus, scholars of the Empiric School of Medicine (330 BCE–ca 400 AD) dismissed the study of physiology by vivisection on the grounds of its cruelty and clinical irrelevance [2]. Early descriptions also reveal an ambivalence by vivisectors towards animal suffering and a need to justify its practice. Galen preferred using pigs and goats over apes because, during brain dissections, the unpleasant expression of the ape discomfited him [3]. Vesalius recognized the cruelty of vivisection but justified it for what it revealed: his thesis *“De humani corporis fabrica libri septem”* (“On the Fabric of the Human Body in Seven Books”) (1543) features a small capital letter “Q” historiating the dissection of a pregnant bitch. (An historiated initial is an enlarged letter at the beginning of a paragraph that contains a picture.) Whilst conceding that the animal “is cruciata”, i.e., is crucified or tortured, “it allowed the demonstration of the unborn puppies struggle to breathe once the placental blood flow was ended” [4]. In the same publication, an historiated capital “Q” shows a conscious pig—immobilized with chains—undergoing tracheal surgery for the placement of a tube allowing periodic lung inflation, the first convincing account of artificial ventilation [5]. That restraining chains were required to immobilize the animal suggests that Vesalius was unaware that, at this time, Paraclesus was using ether (known as sweet oil of vitriol) to produce analgesia in chickens (although the first edition of *Opera Medico-chemica sive paradoxa* (“On the Field of Medicinal Chemistry or Paradoxes”) which contains an account of this was not published until 1605). In this thesis, Paracelsus proposed that the sweet oil of vitriol:

“...quiets all suffering without any harm and relieves all pain, and quenches all fevers, and prevents complications in all disease.”

[6]

It is unfortunate for both humans and animals that 300 years were to elapse before both innovations, i.e., positive pressure lung ventilation and inhalant anaesthetics, were re-introduced into clinical anaesthetic practice.

Harvey, Descartes and the Oxford Group (1578–1665)

Had they been contemporaries, Paracelsus’ interest in pain relief might have benefitted William Harvey’s early studies of “the heart’s motion” in conscious animals whose chest walls had been surgically removed. Harvey complained that the heart’s action was too fast, a complication which *later* prompted him to use “colder animals, such as toads, frogs and serpents…” or “dying dogs and hogs” [7]. That the excessive heart rate was the inescapable result of undergoing thoracic surgery whilst conscious was not overlooked: O’Meara (1665) argued that, “…the miserable torture of vivisection places the body in an unnatural state and that amid the terrible pains of vivisection all the juices are brought to flow together, thus denying the validity of animal experimentation” [3]. Such concerns may have puzzled French philosopher and vivisector Renee Descartes who believed that animals, “When burnt with a hot iron or cut with a knife their writhing and screaming are like the creaking of a hinge, no more” [8]. This belief emboldened some scientists “with less responsible and reflective minds” [3] to conduct even more gruesome experiments [1] whilst elsewhere, it had the opposite effect and promoted consternation—even amongst established vivisectionists. Johann Brunner, Professor of Medicine at Heidelburg, described the animals he dissected as “the martyrs of the anatomists” [3], whilst members of the Oxford Group, specifically Christopher Wren, Robert Boyle and Robert Hooke, not only recognized the cruelty of vivisection and its adverse effects on scientific data, but began preliminary experiments with intravenous anaesthesia. They also considered the use of opium to alleviate the suffering of experimental dogs [9]. Writing to Boyle on the 10th November 1664, Hooke, having that year completed a thoracic dissection and lung inflation study in a dog, complained:

“I shall hardly be induc’d to make any further trials of this kind because of the torture of the creature but certainly the inquiry would be very noble if we could any way find a way soe to stupify the creature as that it might not be sensible which I fear there is hardly any opiate will performe”.

The reference to opiate use is important because both Wren and Boyle had been conducting observational studies on the use of alcoholic opiate concoctions in dogs for the previous 8 years [9]. In *The Usefulness of Experimental Philosophy* (1663), published one year before Hooke’s regretted thoracotomy study, Boyle described an experiment performed by Wren, who injected a warm solution of opium in sack (a sweet white dessert-type wine) into the vein of a dog’s hind leg:

*“We had scarce untied the dog..., before the opium began to disclose its narcotick quality; and almost as soon as he was upon his feet, he began to nod with his head, and faulter and reel in his pace, and presently after appeared so stupified, that there were wagers offered his life could not be saved”*. (The dog survived).

Perhaps aware that “some narckotic” may have alleviated his animal’s suffering, in 1665 a remorseful Hooke wrote:

“The microscope enables one to look at nature ‘acting according to her usual course and way, undisturbed, whereas when we endeavour to pry into her secrets by breaking open the doors upon her, and dissecting and mangling creatures whilst there is life yet within them, we find her indeed at work, but put into such disorder by the violence offered that we cannot tell if the results are of any significance”,

He thus revealed his prescient belief that the extreme physiological responses to painful vivisection were not representative of the normal state. That, he asserted, was only discernible by non-invasive means, e.g., microscopy [10].

Davy (1778–1829)

In April 1799, Humphry Davy reported his initial findings concerning the inhalation of nitrous oxide (N_2_O) and then embarked upon a series of animal experiments culminating with the publication of, “Researches Chemical and Philosophical: Chiefly Concerning Nitrous Oxide and its Respiration”, in 1800. In this, Davy describes guinea pig experiments and the administration of pure nitrous oxide to a “stout and healthy cat”.

“after 5 min the pulse was hardly perceptible; he made no motions and appeared wholly senseless. After 5 min and a quarter he was taken out … in 8 or 9 min he was able to walk … in half an hour he was completely recovered”.

He frequently inhaled the gas himself, once whilst suffering severe pain from a wisdom tooth eruption after which he wrote:

“As nitrous oxide in its extensive operation appears capable of destroying physical pain, it may probably be used with advantage during surgical operations in which no great effusion of blood takes place.”

[11]

Bentham (1748–1841) and Seturner (1783–1841)

That Davy’s findings remained unadopted in medical practice until 1844 is attributed by Cartwright (1950) to the callousness and unprecedented brutality of the 18th-century Englishman [11]. Such callousness may have fuelled a growing philosophical interest in animal suffering, as exemplified by Bentham’s proposal that animals be treated according to their capacity to suffer. In 1789, he asked:

“The question is not, Can they reason? nor, Can they talk? but, Can they suffer?”

[12]

Paradoxically, Bentham’s utilitarianism necessitated his support of “justifiable” vivisection. In a letter to the editor of the Morning Chronicle, 4th March 1825, he wrote:

“Sir—I never have seen, nor ever can see, any objection to the putting of dogs and other inferior animals to pain, in the way of medical experiment, when that experiment has a determinate object, beneficial to mankind, accompanied with a fair prospect of the accomplishment of it. But I have a decided and insuperable objection to the putting of them to pain without any such view”.

While human beings had been capitalising on the analgesic effects of *Papaver somniferum* derivatives since prehistory, the use of opioids in animals remained largely unreported until 1805, following Sertürner’s isolation of morphine [13]. He tested an aqueous alcoholic extraction of the salt on four dogs and a mouse “that he found wandering in the laboratory”. He gave 6 grains to a dog, followed an hour later by another 6 grains. The dogs vomited, had convulsions, and were sleepy, but did not sleep. One “gentle little dog” died. He reported the results of his animal studies in the *Journalder Pharmacie fuer Aerzte und Apotheker* in 1806, a reading of which would probably not have convinced anyone of the new compound’s analgesic potential.

Magendie (1783–1855)

**Figure 1:**
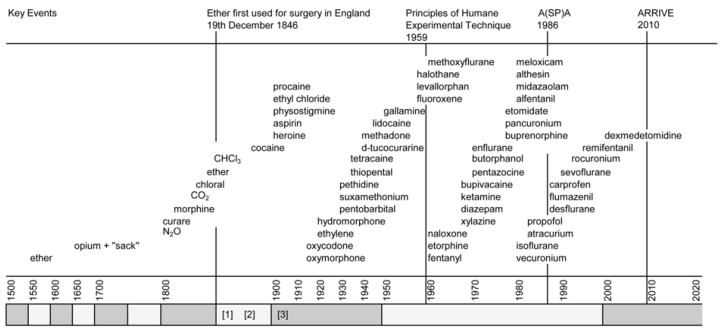
Dates of anaesthetic and analgesic drug discoveries in relation to key events in the refinement of animal experimentation. Alternatively shaded x axis blocks each represent a 50 year epoc. A(SP)A 1986: The Animals (Scientific Procedures) Act 1986. ARRIVE: Animals in Research: Reporting in Vivo Experiments; Reporting guidelines. Additional dates for reference: [1]: 1873 Sanderson publishes, “Handbook for the Physiological Laboratory”; [2]: the Magnan “affair”; [3]: the brown dog affair. CHCl_3_: chloroform.

Francoise Magendie was to become the most influential vivisectionist of all time although when he first began dissecting conscious animals is unknown. Elliott (1987) puts it within a few years of his first publication (1809) because the essay did not describe vivisection, only an intent to conduct it [14]. By the time of his death in 1855, Magendie had been dubbed the “father of experimental physiology” and had done more to foment anti-vivisectionist sentiment in the UK than any other “scientist” in Europe [15]. Descriptions of his vivisections, often performed publicly, abound and reveal absolute indifference to animal suffering and to those who judged his actions cruel. Admittedly, there was a lack of effective anaesthesia—at least for the first forty years of his career—but not the final nine (see Figure 1 for the approximate dates when anaesthetics and analgesics became available for use in animal experiments). France’s first successful ether anaesthetic was administered on 22 December 1846, with reports on its general and obstetrical use being published in January and February 1847, respectively [16]. Magendie himself examined the effects of rectal ether and morphine in dogs [17]. Furthermore, as the first Professor of Physiology at the College de France and Vice-President of the Academie des Sciences, it seems improbable that Magendie could have remained ignorant of the efforts of French physiologists and pharmacologists, e.g., Gerdy, Longet, Flourens, Figuier and Soubeiran to investigate and improve anaesthetic methods [16]. To an astute scientist, the advantages of ether (and later chloroform) in at least some animal experiments should have been clear—and if not for the animals’ benefit—for his own and those of his like-minded mentees, against whom the charges of disgraceful cruelty were being hurled with increasing ferocity from a growing European anti-vivisectionist movement. Magendies defence was anthropocentric: he experimented on animals because he did not wish to experiment on humans. In response to a Quaker who challenged him to:

“......desist from these experiments, because thou hast not the right to cause animals to die or make them suffer....”

Magendie rejoined that if his experiments did not serve humanity, they would indeed be cruel. However, to use animals in order to make discoveries useful to medicine did not merit such reproach [18]. This position applied to anaesthetics. As early as 1847, Magendie had observed that “intoxication caused by sulphuric ether was “little understood” and that only when it was thoroughly understood could one safely and with a clear conscience apply it to man” [19].

Hickman (1800–1830)

Magendies’ animals may have suffered less had Henry Hill Hickman been more persuasive when he visited France in April 1826 to present his ideas on surgical anaesthesia to King Charles Xth. Hickman chose CO_2_ to examine his concept of “suspended animation” with the unique a priori study objective of inducing reversible unconsciousness in order to facilitate surgery [20]. Consequently, Hickman went beyond inducing hypercapnic coma (as early as the 1820s) by testing CO_2_’s ability to produce surgical anaesthesia in dogs and mice. In one of his first experiments, a one-month-old puppy was enclosed beneath a glass cover (bell jar) and

“in ten Minutes he showed great marks of uneasiness, in 12 respiration became difficult, and in 17 Minutes ceased altogether, at 18 Minutes I took off one of the ears, which was not followed by haemorrhage, respiration soon returned and the animal did not appear to be the least sensible to pain, in three days the ear was perfectly healed”

[21]

Hickman’s subsequent pamphlet entitled: “A letter on Suspended Animation containing experiments showing that it may safely be employed during operations on animals with the view of ascertaining its probable utility in surgical operations on the Human Subject,” was ignored by the Royal Society probably because Hickman’s chosen sponsor lacked the necessary enthusiasm for its fair promotion. Furthermore, an 1826 article in The Lancet authored by “Antiquack” (purportedly a professionally envious Davy) entitled ‘Surgical Humbug’ ruthlessly criticized his work and prompted his defection to France, where, despite the support of Napoleon‘s field surgeon, he met a similar response. Since his premature death in 1830, this and previous failures to recognize the potential of his work have been the subject of anguished review [22] because it meant Hickman’s prescient desire to develop surgical anaesthesia in humans took another 20 years for others to achieve.

Martin (1754–1834)

**Figure 2:**
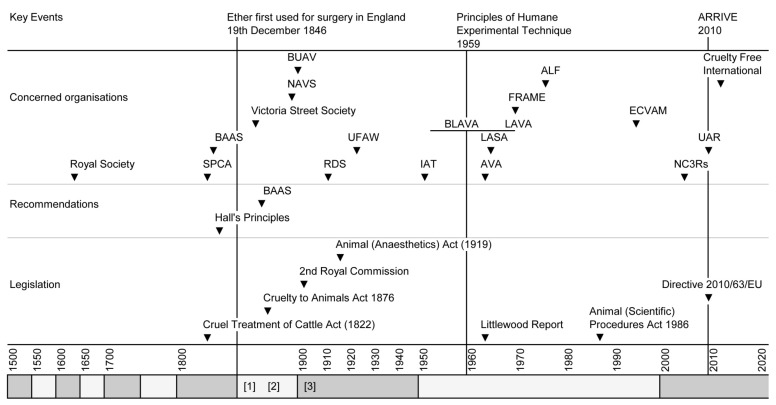
Key events in animal experimentation in the UK partitioned into the introduction of legislative and optional (recommended) constraints, and the formation of societies that have influenced the use of anaesthetics and analgesics in animal experiments. Alternatively shaded x axis blocks each represent a 50 year epoc. A(SP)A 1986: The Animals (Scientific Procedures) Act 1986. ARRIVE: Animals in Research: Reporting in Vivo Experiments; Reporting guidelines. Additional dates for reference: [1]: 1873 Sanderson publishes, “Handbook for the Physiological Laboratory”; [2]: the Magnan “affair”; [3]: the brown dog affair. ALF: Animal Liberation Front; AVA: Association of Veterinary Anaesthetists; BAAS: British Association for the Advancement of Science; BLAVA: British Laboratory Animal Veterinary Association; BUAV: British Union of Anti-vivisectionists; ECVAM: European Centre for the Validation of Alternative Methods; FRAME: Fund for the Replacement of Animals in Medical Experiments; IAT: Institute of Animal Technology; LASA: Laboratory Animal Science Association; NAVS: National Anti-Vivisection Society; NC3Rs: National Centre for the 3Rs; LAVA: Laboratory Animal Veterinary Association; RDS: Research Defence Society; SPCA: Society for the Prevention of Cruelty to Animals; UFAW: Universities Federation for Animal Welfare; UAR: Understanding Animal Research.

Richard Martin MP in conjunction with Edinburgh-born Lord Erskine succeeded in getting the “Cruel Treatment of Cattle Act 1822” or ‘Martin’s Act’, passed into British law—the first legislation against animal cruelty introduced by means of a Parliamentary procedure anywhere in the world. It received Royal assent on the 22 July 1822. Martin’s Act sought to prevent the cruel and improper treatment of horses, mares, geldings, mules, asses, cows, heifers, steers, oxen, sheep and other cattle. The introduction of this and subsequent legislation is shown in Figure 2 (legislation).

Two years later, on 16 June 1824, a meeting was called in Old Slaughters Coffee House, Martins Lane, London at which various clergymen and anti-slavery campaigners launched the Society for the Prevention of Cruelty to Animals (SPCA). Martin was also present and tasked to investigate the markets and streets of the Metropolis, slaughterhouses and the behaviour of coachmen. In 1840, Victoria I granted the “Royal” prefix, and so the RSPCA was born. Neither Act nor Royal Society were initially motivated to control animal vivisection although this was soon to change. The foundation of this and other organisations concerned with vivisection is shown in Figure 2.

Hall (1790–1857)

Marshall Hall, an English neurophysiologist and contemporary of Magendie, pioneered animal welfare from within science. In 1831, he proposed that physiologic procedures be regulated in a way that took into consideration the suffering of animals [19]. He called for the formation of a society for physiological research, which would regulate animal experimentation, for he said, “every experiment… is necessarily attended by pain or suffering of a bodily or mental kind”. In 1835, in his “Principles of Investigation in Physiology,” he outlined five principles to govern animal experimentation, the fourth of which stated (in the days before anaesthesia) that “justifiable experiments should be carried out with the least possible infliction of suffering, often through the use of lower, less sentient animals, such as frogs and fish or even newly dead animals”. (The other points were that: (1) an experiment should never be performed if the necessary information could be obtained by observation; (2) no experiment should be performed without a clearly defined and obtainable objective; (3) scientists should be well-informed about the work of their predecessors and peers to avoid unnecessary experimental repetition; (4) *vide supra*; and (5) every experiment should be performed under circumstances that would provide the clearest possible results, thereby diminishing the need for repetition of experiments.)

Morton (1819–1868), Glover (1815–1859), Simpson (1811–1870) and Snow (1813–1858)

The birth of medical anaesthesia based upon N_2_O and ether in the United States did not depend on animal experimentation, although Morton tested ether on animals [23] and then patients, before administering a convincing anaesthetic at the Massachusetts General Hospital on 16 October 1846.

In the UK, Simpson’s eventual “discovery” and promotion of chloroform in 1847 [24] relied as much on what could be remembered after self-administration as it did upon his own animal studies. That said, Simpson’s pioneering had been eased by others establishing the compound’s pharmacological properties through animal testing—namely Robert Glover, whose studies of bromine, iodine and chlorine compounds, including chloroform and dichlorethane culminated in a thesis entitled ‘On the Physiological and Medicinal Properties of Bromine and its Compounds’. Published in 1842, this was the first to describe chloroform’s anaesthetic effects. Among various experiments, Glover injected 30 and 60 minims (1.6 and 3.6 mL) of chloroform into the jugular vein of two dogs, causing immediate unconsciousness, loss of the eyelid reflex, insensitivity of the paws to painful stimuli and marked motor weakness, a state from which the dogs quickly recovered. Glover did not test chloroform by inhalation, although he described its smell on his animals’ breath [25].

Despite Simpson’s inclination to test newly discovered halogenated organic molecules on himself, it is probable that animal testing saved his life. Having recently synthesized ethylene dibromide, Wemyss Reid (the Professor of Chemistry at the University of Edinburgh) suggested to Simpson that it should be tested on rabbits first. Simpson had intended to inhale ethylene dibromide himself but surrendered the opportunity on discovering the two rabbits so treated had died overnight. It was subsequently discovered that the inhalation of ethylene dibromide causes pulmonary congestion; it is currently used as a soil fumigant to kill nematode worms [26].

John Snow—Simpson’s Yorkshire-born contemporary—was described by Waters (1936) as “the first anaesthetist” because his research spanned basic science and clinical medicine and he answered fundamental questions with respect to anaesthetic safety [27]. Snow also recognised the importance of accuracy in anaesthetic delivery and patient monitoring. Investigating ether (and later chloroform), Snow performed numerous studies on animals, including birds and fish, in which his renowned attention to detail—at least on one occasion—lapsed. Whilst demonstrating ether anaesthesia on a thrush to an audience of Military Surgeons, he became distracted and killed the bird [28]. Snow’s published description of the event reveals the legitimacy of Waters’ accolade.

“This thrush was only in the vapour for about a minute, and it is dead. It had ceased to breathe before I took it out of the jar. It is a result I did not intend, and it has arisen from my going on with the lecture, and looking at my notes, instead of directing my whole attention to the animal”.

Wakley (1795–1862)

On the 19th March 1847, *The Times* reported the death of Ann Parkinson two days after ether administration [29]. Chloroform’s first UK victim was Hannah Greener, who died during induction on 28th January 1848. To assuage growing public concern with the safety of anaesthetics, large-scale animal studies were conducted [30]. In 1848, Wakley reported a study comparing the effects of ether and chloroform on 100 animals—predominantly dogs, but also cats, rabbits, rats, mice, guinea pigs, a hedgehog, a pig, two sheep, a donkey, two mares and some pigeons. From the findings that death resulted in 11 out of 32 animals (34%) anaesthetized with ether and 30 out of 67 animals (44%) receiving chloroform, Wakley concluded:

“…assuredly the more dangerous one of the two would be found in the vapour of chloroform”.

[31]

However, Youngson (1979) emphasized the studies’ hopelessly unscientific character [22], e.g., of the 18 dogs studied, only one received ether (and of the 17 dogs given chloroform, only 4 died (24%)). Whilst Wakley’s conclusions were based on unsound data, they were nevertheless to prove correct.

Bernard (1813–1878)

Claude Bernard, the “father of modern experimental medicine”, died a revered scientist. His attitude to the use of animals and anaesthetics in physiological research was influenced by Magendie under whom he studied from 1834 to 1843. Unlike Magendie, Bernard could not claim ignorance to justify the exclusion of anaesthetics from most—but not all—of his animal studies [19]; nor could he claim adherence to the vitalist principle which he described as being, “entirely contrary to science itself”. Bernard’s considerable contributions to anaesthesia [32] were anthropocentric and achieved at cost to the animals involved. He studied vasomotion—the nervous control of blood vessel diameter—by cutting projections from the stellate ganglion in unanaesthetized rabbits (so discovering cervical sympathetic block and its effects on the eye). His most influential and controversial work—on curare—began in 1844. (Curare is a drug derived from tree bark and applied to blow-dart tips used by South American Indians to paralyse and capture animals. Curare prevents muscle contraction and stops all movement, including breathing. However, it does not affect the central nervous system and so darted animals suffocate whilst being fully conscious. If breathing is artificially supported, a curarised animal can be subjected to surgery and, although fully aware of the process, is unable to move—or vocalize—in agonized protestation.) Having demonstrated its neuromuscular blocking effect in frogs, he used it in dogs to control strychnine-induced convulsions. After further ranine studies, Bernard suspected that death following curare was caused by asphyxia and not loss of consciousness [33]. The realisation that it did not render the animal insentient prompted him to reflect:

“in the animals, one can judge sensitivity only by motor manifestations. Man alone, on recovering from poisoning by curare, would be able to say, supposing that he had retained the memory, whether or not he had suffered”.

He wrote eloquently, and more than once, on his impression of death following curare administration:

“Within the motionless body, behind the staring eye, with all the appearance of death, feeling and intelligence persist in all their force. Can we conceive of a suffering more horrible than that of intelligence present, after succumbing, one by one, of all the organs which are destined to find themselves imprisoned alive within a cadaver?”

This concern did not prevent Bernard from continuing to use curare rather than anaesthetics in his experiments. Furthermore, he promoted its use in experiments to other physiologists. An 1864 essay asserts that the ‘poison becomes an instrument which dissociates and analyses the most delicate phenomena of the living machine’ [34]. Consequently, vivisectors discovered that used in the right quantities, curare made invasive procedures much easier and the drug became a common experimental tool in physiological practice.

Alfort (1846–1863)

For some 17 years, complaints and petitions had been made to various French authorities about the vivisection of horses at the Veterinary College at Alfort, Paris. *The Times* of 8 August 1863 reported that,

“…at Alfort a wretched horse is periodically given up to a group of students to experimentalize upon. They tie him down and torture him for hours, the operations being graduated in such a manner that sixty and even more may be performed before death ensues”.

British Veterinary Surgeons joined elements of the French Press to demand reform. The attack of the *British Medical Journal* was based on the exclusion of anaesthetics:

“It has never appeared clear to us that we are justified in destroying animals for mere experimental research under any circumstances; but now that we possess the means of removing sensation during experiments, the man who puts an animal to torture ought, in our opinion, to be prosecuted”

The beneficial role of anaesthetics during noxious animal experiments was being increasingly recognized. Responding to the Alfort affair, the RSPCA offered a £50 prize for the best essay received on the subject of vivisection. Dr Markham, Physician to Saint Mary’s Hospital London, and Mr Fleming, Veterinary Surgeon to the Third Hussars, won prizes for essays recommending the use of anaesthetics in experiments [15].

Cobbe (1822–1904) and Schiff (1823–1896)

As one of the 19th century’s most effective anti-vivisectionists, the Anglo-Irish reformer Frances Power Cobbe had a major effect on ensuring anaesthetics became a legal requirement in animal experiments in the UK. Prompted by the Alfort scandal, she published, *“The Rights of Man and the Claims of Brutes”* in 1863. In the same year, she petitioned Moritz Schiff “to spare his animals as much pain” as possible. In response, Schiff offered that all his animals had anaesthetics. This may have been true: unlike most of his French and German peers in physiology, Schiff recognized that there

“was a real problem in reconciling the needs of science with the most refined humanitarian sentiment”

and that

“Should the physiologist make use of live animals, he had to suspend their sensitivity, by means of opium, ether or chloroform, depending on which vital functions he wanted to examine and thus to maintain as normal”

[35]

Burdon-Sanderson (1828–1905) [35]

The concern of other British physiologists with the suffering experimentation caused eventually prompted the British Association for the Advancement of Science to publish four recommendations in 1871, two of which promoted the use of anaesthetics, i.e.,

(1) No experiment that can be performed under the influence of an anaesthetic ought to be done without it.
(2) No painful experiment is justifiable for the mere purpose of illustrating a law or fact already demonstrated; in other words, experimentation without the employment of anaesthetics is not a fitting exhibition for teaching purposes.

This view was not entirely consensual. In 1873, John Burdon-Sanderson’s Handbook for the Physiological Laboratory, “a practical description of experimental procedures” was published, providing Cobbe’s anti-vivisectionists with evidence that scientists were indifferent to animal suffering. The handbook made no explicit reference to anaesthesia or how and when to use it in experiments. However, it did index curare, as several contributing authors recommended its use to keep animals still as an alternative to tying them down. The drug’s inability to affect consciousness had been known for approximately 20 years.

Magnan (1847)

Eugene Magnan, another Magendian student, enraged delegates at the 1874 British Medical Association’s congress by injecting alcohol and absinthe into two dogs. The first animal became “dead drunk” while the second had an epileptic seizure. Both later died [36]. The event had at least three consequences. It: (1) made members of the medical profession prosecutable under an amended form of Martin’s 1822 Act; (2) revealed to both anti-vivisectionists and scientists that a law dealing specifically with experimental animal protection was required; (3) re-animated Cobbe’s anti-vivisectionism [15]. It also involved an attempted demonstration of intravenous anaesthesia which failed.

Henniker and Playfair (1875)

Cobbe, working with the RSPCA and indirect support from Queen Victoria, lobbied the Government to legislate against vivisection. In May 1875, the Henniker Bill for Regulating the Practice of Vivisection was introduced to Parliament [37]. The Bill proposed—amongst other things—that “recovery (from anaesthesia) experiments required special approval and that anaesthetics be used in all experiments, excepting those undertaken by individuals with a personal license”. One week later a second Bill representing the scientists’ views was read. This, the Playfair Bill, proposed the regulation of painful animal experiments but recommended the legalisation of painless experiments, including those conducted under anaesthesia. Licenses for painful experiments undertaken without anaesthesia could be granted on several grounds, including “when the use of anaesthesia interfered with the experiment”. The two Bills were in unexpected accord over the general undesirability of painful experiments but sufficiently contradictory to prompt the appointment of a Royal Commission, the creation of the World’s first anti-vivisection society and the establishment of the *Association for the Advancement of Medicine by Research*.

The subsequently appointed Royal Commission of Enquiry spent less time in discussing the undesirability of painful experiments—over which there was some consensus—than in deliberating whether curare was an anaesthetic. The final recommendation was that curare would not be considered an anaesthetic by law, but the rest of the Commission’s findings satisfied neither side who set about drafting new Bills.

Hoggan (1837–1891)

George Hoggan had worked in Bernard’s laboratory in Paris and published an “extraordinarily powerful” letter in the London *Morning Post* on the 2 February1875 describing Bernard’s underuse of anaesthetics and over-use of curare. He wrote: “We sacrificed daily from one to 3 dogs, besides rabbits and other animals, and after four years’ experience, I am of the opinion that not one of those experiments on animals was justified or necessary” [38]. Frustrated with the Commission’s rate of progress, Cobbe, George Hoggan and others formed the “Victoria Street Society for the Protection of Animals from Vivisection” on 2 December 1875 [39]. Initially a non-abolitionist society, it aimed to protect laboratory animals by regulation. Along with the seventh Earl of Shaftesbury, the new society formulated a second Bill. The Cruelty to Animals Act [40] reached the statute book on 15 August 1876 and required that:

“animals must during the whole of the experiment be under the influence of some anaesthetic of sufficient power to prevent the animal feeling pain”

and that

“if the pain is likely to continue after the effect of the anaesthetic has ceased, or if any serious injury has been inflicted on the animal, it be killed before it recovers from the influence of the anaesthetic which has been administered”;

Despite the opposition of the scientific lobby, the new Act also clarified that “the substance known as urari or curare shall not for the purposes of this Act be deemed to be an anaesthetic”.

The Act radicalized the dissatisfied anti-vivisection movement [41]. In 1878, the Victoria Street Society declared its goal of total abolition of vivisection prompting the resignation of moderates like George Hoggan. As opposition to vivisection and Victoria Street Society membership grew, the Society reconfigured and in 1897 became the National Anti-Vivisection Society (NAVS). The following year the NAVS voted to accept humane animal experimentation in the short-term whilst remaining committed to a long-term abolitionist goal. Cobbe promptly left to form the British Union for the Abolition of Vivisection (BUAV) which demanded the total and immediate abolition of animal experiments, which, as Cruelty Free International, it still does.

Koller (1857–1944) and Corning (1855–1923)

Karl Koller initially tested cocaine hydrochloride to the cornea of rabbits and dogs in 1884 and reported that, after one-half to one minute, “insensitiveness” was complete and lasted ten minutes [42]. In 1888, Koller moved to the United States and practiced ophthalmic surgery in New York. Here, the neurologist James Corning injected 20 minims (1.3 mL) of a 2% cocaine solution into the space between two inferior dorsal vertebrae of a young dog [43]. Within 5 min, he noted incoordination and later, weakness and anaesthesia of the animal’s hind quarters which resolved completely in approximately 4 h. Local anaesthetic techniques had been discovered.

Hobday (1869–1939)

The veterinary surgeon Sir Frederick Hobday arguably established veterinary anaesthesia as a specialty for purposes beyond the laboratory. In 1906, he published “Surgical diseases of the dog and cat: with chapters on anaesthetics and obstetrics”. Chapter 4, entitled, “The administration of anaesthetics” was 23 pages long, with five pages devoted to local anaesthesia, 16 pages to general anaesthesia and two pages to morphia and chloral (hydrate) [44]. In 1915, Hobday published the first book dedicated to the subject, “Anaesthesia & Narcosis of Animals and Birds”. In describing the use of chloroform, ether, ACE mixture (alcohol, chloroform, ether) ethyl chloride and nitrous oxide for use in horses, oxen, sheep, goats, pigs, dogs, cats, monkeys, wild and semi-domesticated animals and birds, Hobday provided information of potential value for improving laboratory animal anaesthesia.

Starling, Bayliss, Dale, Coleridge, Lind-af-Hageby and the brown dog (1902–1910) [45]

Starling (Professor of Physiology at University College London) conducted pancreatic surgery on a small brown terrier in December 1902. He re-opened the dog’s abdomen in February 1903 before a class of medical students. On completing the second operation, Starling handed the animal to Bayliss, who, in making a new wound, contravened the “no re-use” principle extant in the 1876 Act. During the third operation, the animal “suffered greatly” and made purposeful movements, indicating inadequate anaesthesia. The dog was finally given to Henry Dale, an unlicensed research student, who killed the animal. Louise Lind-af-Hageby and Leisa Schartau observed and recorded the events in, *“The Shambles of Science: Extracts from the Diary of Two Students of Physiology”.* Stephen Coleridge, NAVS secretary, read the book and after recognising breaches of the 1876 Act, made a public statement against Bayliss, who issued a libel suit. Bayliss won the ensuing case but Coleridge won the public: £5735 was collected and partly paid for a statue of the brown dog which was unveiled in Battersea Park on 15 September 1906. Medical students vandalized the statue and it became the focus of fighting between medical pro-vivisectionists, the police, anti-vivisectionists and the locals—who had developed a fondness for the effigy. The statue’s removal on 10 March 1910 provoked further anger: nine days later, more than 3000 people marched from Hyde Park Corner to Trafalgar Square, where a public meeting was held. Politically relevant public unrest had originated from the mismanagement of the small dog’s anaesthetic.

Public reaction to the brown dog affair encouraged the appointment of a second Royal Commission on Vivisection in 1906. Amongst other matters, Stephen Coleridge proposed that the use of curare should be entirely prohibited in animal experiments. The Commission eventually recommended that the use of curare in experiments required special certification, that animals in such experiments must be anaesthetized before the operation and kept anaesthetized until death. They also recommended that a Home Office appointee should be present during experiments in which curare was used.

Paget (1855–1926) [46]

**Figure 3:**
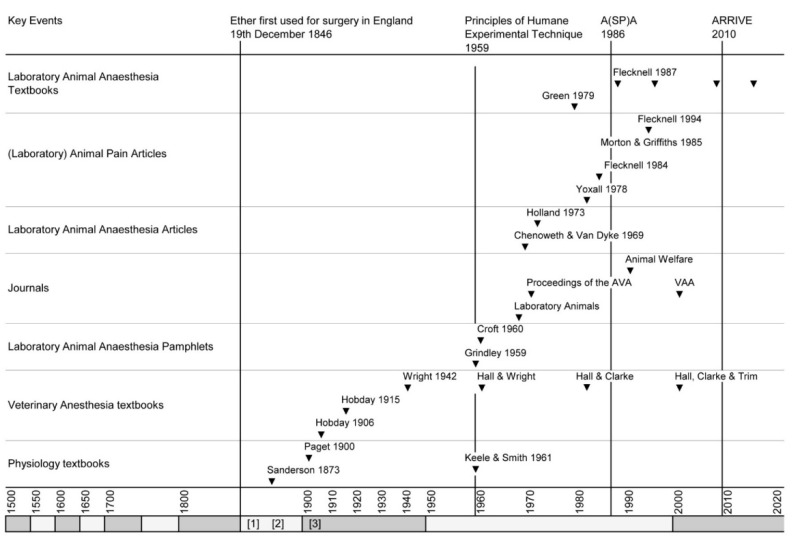
Dates of the publication of material contributing to promotion of anaesthetics and analgesics in animal experiments. Alternatively shaded x axis blocks each represent a 50 year epoc. A(SP)A 1986: The Animals (Scientific Procedures) Act 1986. ARRIVE: Animals in Research: Reporting in Vivo Experiments; Reporting guidelines. Additional dates for reference: [1]: 1873 Sanderson publishes, “Handbook for the Physiological Laboratory”; [2]: the Magnan “affair”; [3]: the brown dog affair. AVA: Association of Veterinary Anaesthetists; VAA: Veterinary Anaesthesia and Analgesia. Grindley [47] Keele and Smith [48] and Chenoweth and Van Dyke [49] are difficult to locate and on 12 September 2020, were unavailable online. Morton and Griffiths [50] is a landmark paper insofar that it was the first to emphasize an ethical and scientific imperative to use analgesics in laboratory animals.

The publics’ perception of scientists versus anti-vivisectionists began shifting after the brown dog affair because a link between scientific growth and an improving quality and quantity of life was becoming apparent. Medical scientists, becoming sensitive to the public’s concerns, were also making efforts to dispel the accusations of cruelty being made against them whilst capitalising on the role of animal experimentation in understanding—if not treating—conditions such as diabetes. This was exemplified by the publication of Stephen Paget’s *Experiments on Animals* in 1900. A tome of 381 pages, 24 were devoted to the chapter on “Anæsthetics Used For Animals”—a considerable improvement from Sanderson’s aforementioned handbook in which advice on anaesthesia was notable by its absence. Please see Figure 3 for the dates of the publication of material contributing to the promotion of anaesthetics and analgesics in animal experiments.

Wright (1897–1971)

Hobday’s publications were superseded by (J.G.) Wright’s Veterinary Anaesthesia, which was first published in 1942. Its objective was to serve as a student textbook and as a reference manual for veterinarians in practice. To this end, the book in all its editions served its purpose well. However, laboratory animal anaesthesia was never prioritized, e.g., the current (11th edition) edited by Hall (deceased) Clarke and Trim, covers most of the laboratory animal species albeit subsumed in a chapter entitled “Anaesthesia of zoological species (exotic pets, zoo, aquatic, and wild animals)”. An examination of Figure 3 (below) reveals that the separation of an increasingly specialized laboratory animal anaesthesia literature from that describing the common domesticated species began with Hall and Wrights 5th edition (1961).

Hume (1886–1981) Russell (1925–2006) and Burch (1926–1996) [51]

In 1926, Charles Hume founded the University of London Animal Welfare Society which became the Universities Federation for Animal Welfare (UFAW) in 1938. Its goals “were to ‘tackle animal problems on a scientific basis, with a maximum of sympathy but a minimum of sentimentality”. Amongst its formal aims were to enlist the influence of university men and women on behalf of animals, wild and domestic and to lessen, by methods appropriate to the special character of a university organisation, the pain and fear inflicted on animals by man. In 1954, Hume appointed William Russell and Rex Burch and inaugurated a systematic study of the ethical aspects of experimental animal techniques. Their report, published in 1959 and entitled “*The Principles of Humane Experimental Technique*”, condensed humane techniques into three categories; replacement, reduction, and refinement—the 3Rs. Replacement involved using reliable non-animal methodologies when they existed. When they did not, reduction meant using the least number of animals to achieve scientific objectives. Refinement referred to any measure improving the welfare and experiences of animals that could not evade experimentation by being replaced or surplus to study requirements. In discussing refinement and anaesthesia Russell and Burch state:

“the most generally important of all is that of anaesthesia, the supreme refinement procedure. This has occasioned perhaps the greatest single advance in humane technique, and has at the same time been virtually indispensable for the advance of experimental biology”.

[52]

Russell and Burch’s appreciation of anaesthesia can be condensed into four general themes (1) its importance, arising from its ability to eliminate pain and suffering; (2) concerns with administration, i.e., doses and timing; (3) the hazards of neuromuscular blocking agents; (4) the promotion of local anaesthetic techniques.

Croft (1919–2009)

Neither Russell nor Burch were animal anaesthetists and throughout their “Principles” refer—when necessary—to the experimental work of Phyllis Croft, a veterinary neurologist. For example,

“Croft has also recently (1957) discussed the condition for veterinary and experimental use of the relaxants or curariform drugs which block neuromuscular transmission among other effects and which in general should only be used in conjunction with general anaesthesia and in mammals, facilities for artificial respiration”.

[53]

In 1960, Croft, in conjunction with UFAW, published, “An introduction to the anaesthetics of laboratory animals”. The booklet, which was 31 pages long and written for technicians and junior graduates who had no previous experience of anaesthesia, described injection technique and the choice of anaesthetic, and contained sections on practical anaesthesia in rabbits, guinea pigs, hamsters, rats and mice. The selection of drugs was confined to ether, thiopentone and pentobarbitone, and its emphasis was on simplicity [54]. Its publication represents the beginnings of a literature devoted to laboratory animal anaesthesia.

Littlewood (1965)

By 1963, the number of animals involved in experiments was counted in millions while the Cruelty to Animals Act (1876) had remained largely unchanged [55]. A Departmental Committee on Experiments on Animals, chaired by Sir Sidney Littlewood was assigned “to consider the present control over experiments on living animals, and to consider whether, and if so what, changes are desirable in the law.” Published in 1965, the Littlewood Report finally established an uncompromising recognition that, “the use of muscle relaxing drugs which, in effect, renders an animal physically helpless whilst leaving it fully conscious, opens the way to experiments of extreme cruelty.”

Singer (1974)

Numerous factors, including two world wars, a major economic recession and the fear of nuclear annihilation distracted public attention from animal experimentation for much of the 20th century. However, anti-vivisectionism was re-animated in 1974 when “Animal Liberation” was published by the Australian philosopher Peter Singer. The Animal Liberation Front (ALF), founded in 1976, considered the work to be the founding philosophical statement of its *raison d’etre* and subsequently ensured—through various activities—that the subject of laboratory animal welfare became a matter of public concern. However, the chapter featuring laboratory animals and entitled “Tools for Research” tends to overstate the usefulness of in vitro and ex vivo methodologies, while understating the role of national and institutional controls. It contains harrowing and detailed descriptions of selected animal experiments, mainly conducted by the US military and psychiatric researchers delivering noxious agents and, or stimuli to conscious animals—usually primates or dogs. Importantly, the role of anaesthetics in experimental refinement is poor, or mis-represented. For example, when discussing the role of animals in traumatic shock research, Singer summarizes scientific consensus with:

“They (the scientists) discouraged the use of anaesthesia… the influence of anaesthesia is controversial… and in the reviewer’s opinion prolonged anaesthesia is best avoided.”

[56]

and

“Experimenters may consider it unnecessary to include in their reports any mention of what happens when… animals recover consciousness in the midst of an operation because of an improperly administered anaesthetic…”.

[56]

Singer makes no reference to “The Principle of Humane Experimental Technique”, while the terms “anaesthesia”, “anesthesia” and “analgesia” do not appear in the book’s index. Arguably, a more balanced analysis of the benefits of anaesthetics and analgesics in animal experiments might have reduced the number of criminal acts subsequently committed by the ALF against scientists and their work-places while offsetting the public’s increasing tendency to view scientists as cruel and uncompassionate.

Holland and Yoxall (1973–1978)

Concerns with unfeeling science may have been allayed had Holland’s 1973 article in the Canadian Anaesthetists Society Journal [57] been more widely read. Worried about the paucity of information available for laboratory animal anaesthesia, e.g.,

“…the relatively low standard of veterinary anaesthesia practised, together with the wide variety of animals which are now being anaesthetized in laboratories and veterinary hospitals”

Holland asserted that:

“vertebrate animals (and perhaps some invertebrates too) have similar pain pathways and similar perceptions of pain as man—their lack of ability to communicate does not indicate a lack of awareness of pain and does not condone inhumane treatment”.

Holland goes on to describe her personal recommendations for anaesthetising dogs, cats, rabbits, guinea pigs, infrahuman primates [sic], rats, mice, pigs, ruminants and birds. She also argues that:

“In spite of their low phylogenetic position, common humanity at the very least dictates that they should be anaesthetized prior to surgical procedures”,

so goes on to describe anaesthesia in fish, amphibians, reptiles and invertebrates”. The beliefs expressed in this article in relation to animal pain are remarkable insofar that Holland was a medical anaesthetist with an interest in child health. Furthermore, her sentiments on animal pain were published 5 years before those of Yoxall’s.

Yoxall [58] was an early member of the Association of Veterinary Anaesthetists and although he held the Royal College Diploma in Veterinary Anaesthesia, his major influence was in veterinary clinical pharmacology. His paper, “Pain in small animals - its recognition and control” (1978) was the first paper in the veterinary medical literature to highlight the importance of appreciating pain—albeit in dogs and cats. The article outlined the then current concepts in pain physiology and described the clinical use of analgesics. Taken with Holland’s sentiments, the publication of Yoxall’s paper represents a point in time at which some began recognising the importance of eliminating pain rather than just consciousness in animals undergoing noxious procedures.

Green (1979) [59]

Colin Green, a veterinarian working for the Medical Research Council authored the first book devoted to laboratory animal anaesthesia in 1979. Accurately entitled “Animal Anaesthesia” (the book covered all common laboratory, domestic, wild, and zoological specimens), the book represented a major advance because it condensed a mass of disparate information on the subject. Green was also in a position at the Clinical Research Centre to conduct anaesthetic trials (under the 1876 Act) on species in which little information existed. The book benefitted from Green’s association with Richard Medd, who, as a veterinarian and experimental surgeon working at Huntingdon Life Sciences, commented extensively on the manuscript and provided additional information from his own experiences. “Animal Anaesthesia” did not run to further editions so the numerous references it cites are now out-dated—albeit of considerable historic interest. The book also required some understanding of physiology and pharmacology. The messages Green espoused in his book concerning safe anaesthetic practice remain applicable today.

Flecknell

Paul Flecknell was influenced by Yoxall’s advocacy of analgesic use in animals and in 1984 authored “The Relief of Pain in Laboratory Animals” [60]. This work reviewed the analgesic drugs then available and the experimental evidence for their efficacy in laboratory animals. The information was then extrapolated to the clinical situation to provide guidance as to methods of achieving effective analgesia. The publication was the first to emphasize that anaesthesia did not necessarily ensure freedom from post-operative pain and suffering and that analgesics, as distinct from, and in addition to anaesthetics were required for the optimal refinement of noxious procedures.

This message was continued in a 1994 publication, “Refinement of animal use—assessment and alleviation of pain and distress” which also focused on pain recognition [61]. Warning against the dangers of uncritical anthropomorphism, Flecknell complained that the methods used for the assessment of pain and distress then available were unsatisfactory and appealed for more objective methods. This appeal led to a measureable increase in laboratory animal pain research, not least from the Comparative Biology Centre at the University of Newcastle, where Flecknell was Director.

In recognition of the increasing vintage of Green’s contribution, Flecknell’s “Laboratory Animal Anaesthesia: An Introduction for Research Workers and Technicians” was published by the Academic Press in 1987 [62]. As the full title suggests, these “introductions” were intended to be, and have remained, relatively uncomplicated guides for staff with limited training in veterinary anaesthesia. However, they provide enough information for laboratory workers to be able to safely anaesthetize commonly encountered laboratory animal species. That subsequent editions have appeared in 1996, 2009 and 2015 attests to the book’s appeal.

The advances being made in laboratory animal anaesthesia and analgesia at this time meant that the Animal (Scientific Procedures) Act 1986 (A[SP]A) did not justify *major* changes to the general use of anaesthetics and analgesics in laboratories. Beyond reviewing the licensing system, providing more specific definitions for “regulated procedures” and “protected animals”, and introducing named personnel to ensure compliance at establishment level, the Act required additional personal and project license fulfilments for those intending to use neuromuscular blocking agents. Personal licensees were required to provide evidence that they were competent in managing anaesthetics in the species they intend to paralyse and were conversant with Appendix H (Guidance on the use of neuromuscular blocking agents [NMBAs]) [63].

Between 2005 and 2011, Flecknell and colleague conducted four structured reviews of the biomedical literature [64,65,66,67] which, in recording the reported use of analgesics in laboratory animals in the biomedical literature, acted as an estimate of experimental refinement—at least in terms of analgesic use (see Table 1 for details of these, and related studies) [68]. All studies intimated that analgesics were not being extensively used in noxious experimental procedures, but aware that discrepancies may have existed between the *reported* and *actual* use of analgesics, Richardson and Flecknell (2005) made retrospective contact with the corresponding authors of papers in which analgesics had not been mentioned [64]. Of these, 71% replied that analgesic use had not been reported because they had not been used, revealing the existence of a widespread problem.

Flecknell’s contribution to practical training in laboratory animal anaesthesia continues in the form of e-learning modules which have been reformulated to meet the learning outcomes set out by the EU Expert Working group on Education and Training Framework [69]. The latter was established by the European Commission to develop a common education and training framework for the EU to fulfil the requirements under Articles 23, and 24 of Directive 2010/63/EU on the protection of animals used for scientific purposes.

The burgeoning availability of educational resources and information over the last two decades may be exemplified by the NORECOPA website (https://norecopa.no) which has extensive information on laboratory animal anaesthesia and analgesia as well as a collection of links to international guidelines, information on textbooks, and links to resources for education and training. The website currently runs to some 10,000 pages and cites global resources of relevance to the 3Rs.

Kilkenny, Parsons, Kadyszewski, Festing, Cuthill, Browne, Emerson, Altman and Smith (2010–2020)

Flecknell’s findings [64,65,66,67] supported a growing view that biomedical reportage was inadequate, causing the National Centre for the Replacement, Refinement and Reduction of Animals in Research (NC3Rs), a UK Government-sponsored scientific organisation, to commission a survey to review standards of description in the animal research literature. The subsequent report indicated that serious omissions were present in the way research involving animals was reported [70].

After widespread consultation the ARRIVE guidelines were published. These consisted of 20 items describing the minimum information that scientific publications reporting animal research should include. With respect to anaesthetics and analgesics, three explicit and four potential requirements were listed indicating the relative importance attributed to these in reporting animal experiments [71].

However, despite widespread uptake of the ARRIVE guidelines by journals, funding bodies, universities, organisations and learned societies, it soon became clear that their effect on reporting animal experiments was limited. For example, an examination of 400 scientific articles describing major survival surgeries in dogs, primates, pigs, mice, rats and other rodents for the completeness of the information they provided on anaesthesia and analgesia concluded that the current scientific literature could not be trusted to present full details on the use of animal anaesthetics and analgesics [72]. Another compared the extent to which all elements of the guidelines had been fulfilled in Journals which supported, or did not support the guidelines at time-points before and after guideline introduction, and found that journal support for the ARRIVE guidelines had not resulted in, “a meaningful improvement in reporting quality, contributing to ongoing waste in animal research” [73]. The reported use of NMBs in laboratory pigs in the literature was examined [74] and, based on (1) the absence of information confirming that animals were adequately anaesthetized, and (2) the affirmation by corresponding authors that reported information reflected the actuality, prompted the conclusion that a proportion of laboratory pigs undergoing noxious procedures are likely to be aware under general anaesthesia.

The ARRIVE 2.0 guidelines were introduced in 2020 in an attempt to resolve poor reporting tendencies [75]. However, it had already (2018) been pointed out that the complete description of badly planned and managed experiments would not increase study reproducibility, refine the conditions for, nor reduce the number of animals wasted in such a study [76]. Consequently, the PREPARE (Planning Research and Experimental Procedures on Animals: Recommendations for Excellence) guidelines were developed as one of Norecopa’s (https://norecopa.no/PREPARE) contributions to tackling the reproducibility crisis. It is to be seen whether these and, or ARRIVE 2.0 will affect the implementation and reporting of refinement in terms of providing and describing the use of anaesthetics and analgesics in future animal experiments.

## 2. Conclusions

This historical review of vivisection reveals that anaesthetics and analgesics have had a major beneficial effect on laboratory animal welfare. Scientific concerns that anaesthetic and analgesic drugs may temporarily disperse data and affect scientific conclusions continue to be countered by demonstrating the similar, though adverse and oft-prevailing effects of pain. In any case, the use of analgesics increases the external validity of animal models used in the study of noxious human conditions: that animal models of painful human conditions should be deprived of the care that the modelled human could expect is fundamentally unjust and scientifically unsound.

Persistent philosophical objections to the use of animals in research are accommodated by the replacement principle, which aims to achieve the eventual exclusion of all animals from all experiments. The funding and promotional activities of Fund for the Replacement of Animals in Medical Experimentation (FRAME) and the NC3Rs attest to the sincerity of this aim. However, public objections—which have historically been based on the argument that animal research is cruel (and pointless)—are immediately addressed by the refinement afforded by terminal anaesthetics, or appropriate anaesthetic and analgesic techniques in recovery studies. Repeated Ipsos Mori polls reveal that societies’ majority acceptance of animal experimentation—at least in the UK—is based on assurances that it is for medical research purposes, that there is no alternative and that there is no unnecessary suffering [77]. The latest poll also revealed a public belief that laboratory animal veterinarians continue to be the most trusted source of information on animal research. It follows that the publics’ general concerns with the use of animals in research may be further allayed were qualified veterinary specialists, e.g., Diplomates of the European (or American) Colleges of Veterinary Anaesthesia and Analgesia, to become increasingly involved in assisting their colleagues in laboratory animal medicine when noxious animal experiments are planned.

Despite *some* success in replacing and reducing the numbers of animals used in research, the constant introduction of new research methodologies, e.g., gene editing, imaging, et cetera, and the requirement for new diagnostic or therapeutic devices to undergo pre-clinical safety and efficacy testing in animals, makes continuous demands on those concerned with experimental refinement. Arguably, this may apply less to anaesthesia. The compulsory training of laboratory animal personnel in the safe delivery of modern anaesthetic techniques, combined with the widespread availability of information, has reduced the need for any urgent change—at least in the management of non-invasive, non-painful and straightforward procedures. This does not apply to the use of analgesics, particularly when noxious recovery procedures are involved, because considerable gaps persist in the field of laboratory animal pain recognition and treatment. Some have suggested that this justifies the need for prospective pain studies [78]. However, the point has already been made [79] that refinement principles are better served by improved reporting. The original ARRIVE guidelines (2010) requested that articles describing animal experiments provide: (1) precise details of how anaesthetics and analgesics are used; (2) a description of welfare-related assessments and interventions; and (3) that all details of adverse events, e.g., unmitigated pain, be described, and that any modifications made to experimental protocols in order to reduce adverse events are revealed, e.g., the provision of effective analgesia. Given that these requirements have not been forthcoming, it has been suggested that amongst ARRIVE-subscribing journals (and others) a more determined editorial enforcement of the guidelines would go a long way to increasing the information available on pain management in laboratory animals [79]. Unfortunately, the ARRIVE 2.0 guidelines have relegated, “interventions taken in experimental protocols to reduce pain, suffering, and distress” and “reports of expected or unexpected adverse events” to the “Recommended Set” rather than the obligatory “Essential 10”. It remains to be seen what effect this will have on the role of anaesthetics and analgesics in the ongoing refinement of animal experiments.

## Figures and Tables

**Table 1:** The Extent of anaesthetic and analgesic administration in animal experimentations as determined by literature analysis. NA: not applicable.

Publication Year	Species	Sampling Period	n Articles	% Analgesic Use Reported	% Anaesthetic Use Reported; Ranked Use of Different Techniques	Citation
2005	Rodents	1990–1992	112	2.7	NA; ether > “other” > pentobarbital > injectable combinations	[64]
		2000–2002	126	19.8	NA; pentobarbital > injectable combinations > “other” > isoflurane	
2009	Mice	2000–2001	9	7	NA; xylazine/ketamine > pentobarbital > halothane	[65]
		2005–2006	69	9	NA; pentobarbital = xylazine/ketamine > isoflurane	
	Rats	2000–2001	77	31	NA; pentobarbital > xylazine/ketamine > ether > injectable combinations	
		2005–2006	17	37	NA; isoflurane > pentobarbital > injectable combination	
2009	Rabbits	2000–2001	15	40	Anaesthetics not described	[66]
		2005–2006	15	53		
	Pigs	2000–2001	15	67		
		2005–2006	15	67		
	Sheep	2000–2001	15	64		
		2005–2006	15	73		
	Dogs	2000–2001	15	40		
		2005–2006	15	53		
	NHP	2000–2001	15	40		
		2005–2006	15	67		
2011	Rabbits	1995–1997	64	16	Anaesthetics not described	[67]
		2005–2007	63	50		
2016	Pigs	2012–2014	233	83	Isoflurane > not described > propofol	[68]
